# Surgical treatment and long-term outcome of aortic valve endocarditis with periannular abscess

**DOI:** 10.1007/s12471-020-01409-x

**Published:** 2020-03-20

**Authors:** S. I. Croon, A. Angkasuwan, A. H. van Straten, A. Khamooshian, T. W. Elenbaas, M. A. Soliman-Hamad

**Affiliations:** 1 Department of Cardiothoracic Surgery, Catharina Hospital, Eindhoven, The Netherlands; 2 Department of Science, University College Roosevelt, Middelburg, The Netherlands; 3 Department of Cardiothoracic Surgery, Groningen University Medical Centre, Groningen, The Netherlands

**Keywords:** Aortic valve replacement, Aortic root, Endocarditis (all infectious agents), Heart valve

## Abstract

**Objectives:**

Aortic valve endocarditis is occasionally complicated by periannular spreading of the infection and abscess formation, leading to a more aggressive course of the disease and life-threatening complications. This retrospective observational study investigated the long-term outcomes of patients with this complication, which was surgically managed with annular reconstruction and aortic valve replacement.

**Methods:**

Between 1998 and 2018, 69 patients were identified with aortic valve endocarditis complicated by periannular abscess formation. All patients were treated with debridement of the infected tissue, gentamicin filling of abscess cavities, annulus reconstruction with bovine pericardium, and valve replacement. Long-term follow-up was performed to detect the rate of recurrence of endocarditis, aortic valve reoperation and survival.

**Results:**

Mean age was 58 ± 15 years, 81% of patients were male, and the infected valve was native in 51% of all patients. The overall mortality was 36%, with a 30-day mortality of 13% and 120–day mortality of 16%. Five- and 10-year survival was 69.4 ± 12.0% and 55.7 ± 14.3%, respectively. Ten-year freedom from recurrent endocarditis was 83.5 ± 13.3%.

**Conclusion:**

Endocarditis with annular abscess remains associated with high morbidity and mortality and aggressive treatment of the infected tissue and abscess cavities is crucial. Compared with earlier literature, long-term outcome of annular reconstruction in this series is comparable to that of aortic root replacement.

## What is new?

Despite the high morbidity and mortality of aortic valve endocarditis complicated by periannular abscess formation, reconstruction of the annulus with a gentamycin-pericardial patch and valve replacement is a successful treatment. In comparison with earlier reports of the literature, this treatment has a comparable outcome to root replacement therapy.

## Introduction

Active infective endocarditis is a life-threatening condition that commonly affects the heart valves and requires urgent medical care. Appropriate antibiotic therapy is crucial, although surgical intervention is needed in approximately one-third of the cases [1]. Early surgery is recommended for improving short-term as well as long-term survival [2, 3]. Up to 42% of infective endocarditis cases is complicated by periannular spreading of the infection and abscess formation, with a relatively higher occurrence in prosthetic valve endocarditis (PVE) compared with native valve endocarditis (NVE) [4, 5]. While rare, aortic periannular and paravalvular abscess formation are associated with a more aggressive course of the disease, which in turn may lead to life-threatening complications such as sepsis and perforation [6]. Delay of surgery might lead to further destruction of the aortic root, a higher risk of complications, and a more complex surgical procedure. The associated morbidity and mortality rate remains high despite therapeutic advances, indicating the need for more effective interventions [6]. Generally, the primary goal of surgery in these cases is radical debridement of the infected tissue, reconstruction of the excised area, and replacement of the aortic valve or root [7]. The choice of annular reconstruction with aortic valve replacement or aortic root replacement depends on the extent of the annular destruction. While aortic root replacement was the primary choice in multiple recent studies [8, 9], we solely used annular reconstruction and aortic valve replacement in our centre as there is an increased risk of valve deterioration with aortic root replacement [10]. In this study, we sought to review our database since 1998 to investigate the incidence and long-term outcome of patients with endocarditis complicated by periannular abscess, who are treated with annular reconstruction and aortic valve replacement.

## Methods

In the Catharina Hospital in Eindhoven, the Netherlands, 525 patients underwent surgery for infective endocarditis between 1998 and 2017. Sixty-nine patients (13%) who suffered from infective endocarditis of the aortic valve complicated by aortic periannular abscess formation were identified from the Catharina Hospital Eindhoven cardiac surgery database for a retrospective study. To obtain any missing data, we consulted other sources including hospital medical records and operation room reports. The included patients required patch reconstruction and were treated with gentamicin sponges. The local medical ethics committee approved the study and waived the need for an informed consent.

### Operative procedures

The procedures involved the usual type of myocardial protection and cardiopulmonary bypass techniques [11], hence not further specified in this report. After sufficient exposure of the infected area, extensive debridement was performed, including sutures and pledgets in case of prosthetic valve endocarditis. Reconstruction of the aortic root, annulus and other affected areas was then undertaken primarily by using bovine pericardium. Part of the reconstruction is managing the abscess cavities after thorough cleaning. The technique employed in this setting is filling the abscess cavities completely with gentamicin-treated sponges such as Gentafleece (Baxter, Deerfield, IL, USA) or Garacol (EUSA pharma, EU) and closure of the cavity with bovine pericardial patch. If the abscess cavity has eroded the aortic annulus, prosthetics in the aortic position are attached—partly—to the reconstructed area. The aortotomy is then closed.

### Follow-up

Follow-up data were acquired by reviewing patient files in digital and hard-copy format and on film. For patients who were referred for surgery by other hospitals and afterwards returned to these hospitals, we collected data by contacting the referring cardiologists. Patients were approached directly in case of lost to follow-up. They were first asked for their consent before answering the questionnaire. Date of follow-up was July 1st, 2018. Follow-up was complete for all patients. The median follow-up was 3.58 (interquartile range from 1.13 to 9.58) years, ranging from 0 to 20 years.

### Definitions

#### Overall mortality:

defined as all-cause mortality occurring during the follow-up period. It was retrieved through consulting the municipal personal records databases.

#### Re-intervention:

defined as reoperation because of recurrent infective endocarditis.

### Antibiotics policy

Antibiotic treatment of patients with endocarditis usually starts before the culture and susceptibility pattern of the offending microorganism are known. The empirical treatment is focused on the most likely pathogens. Subsequently, when the microbiological data is known, the antibiotics and the duration of treatment are adapted depending on the tissue and blood culture results. The duration of treatments is most commonly six weeks, unless the microbiologist advises another policy. The antibiotic policy is reconsidered when the patient shows no improvement after three days.

### Statistical analysis

Data were analysed using IBM SPSS Statistics 25.0. software (New York, USA). Continuous variables are depicted as mean ± standard deviation or as the median and interquartile range, and categorical variables as numbers (%). Preoperative variables included age, sex, body mass index (BMI), body surface area (BSA), diabetes mellitus, renal failure, hypertension, chronic obstructive pulmonary disease, cerebrovascular accident (CVA), peripheral vascular disease, impaired left ventricular function, and native or prosthetic valve endocarditis. Postoperative complications included re-exploration for bleeding, pulmonary complications, CVA, pacemaker implantation, reinfection, re-intervention, as well as early and late mortality.

The significance level was set at a *p*-value of <0.05. The Kaplan-Meier method was used to calculate estimates of survival and freedom from events. The log-rank (Mantel-Cox) statistic was used to calculate any differences between the survival curves.

## Results

**Fig. 1:**
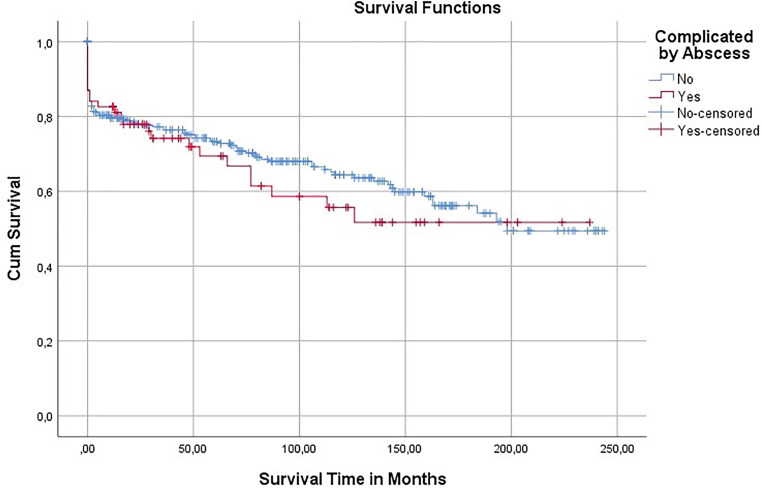
Kaplan-Meier curve: overall survival of the study population

Tab. 1 shows the basic characteristics of the study population. The majority (81%) were male and the mean age was 58 ± 15 years. Using the Kaplan-Meier method, it was estimated that the mean survival time was 143.5 ± 14.32 months. This in comparison with aortic endocarditis patients without abscess formation (Fig. 1). Overall mortality in this series included 25 patients (36%) with a 30-day mortality in 9 patients (13%). In this group, 34 cases (49%) were re-operations, hence prosthetic valve endocarditis and most abscess cavities were located in the annular region (Tab. 2).

**Table 1:** Basic characteristics (*n* = 69)

Baseline characteristics	*N* (%)
Age, years	58 ± 15
Male gender	56 (81)
Body mass index, kg/m^2^	26.4 ± 4.5
Body surface area, m^2^	1.9 ± 0.2
Diabetes mellitus	8 (12)
Renal failure	23 (33)
Hypertension	24 (35)
Chronic obstructive pulmonary disease	5 (7)
Transient ischaemic attack/stroke	9 (13)
Peripheral vascular disease	5 (7)
Impaired left ventricular function:	12 (17)
Native valve endocarditis	35 (51)
Prosthetic valve endocarditis	34 (49)
Emergency	46 (67)
Re-operation	34 (49)

Data are expressed as mean ± standard deviation or number (%)

**Table 2:** Operative data and hospital stay

Operative procedure	*N* (%)
AVR^a^	56 (81)
AVR + CABG	4 (6)
AVR + MVP^a^	5 (7)
AVR + MVR	3 (4)
AVR + CABG + MVP	1 (1)
*Type of valve prosthesis:*	
Mechanical	50 (72)
Bioprosthetic	19 (28)
*Main location of abscess:*	
Left coronary cusp	8 (12)
Right coronary cusp	5 (7)
Non-coronary cusp	18 (26)
Multiple locations	19 (28)
– Commissure involved	3 (4)
– No commissure involved	16 (23)
Only commissural	7 (10)
Other	5 (7)
Unknown	7 (10)
Aortic clamp time, min	110 ± 40
Cardiopulmonary bypass time, min	157 ± 70
ICU stay, days^b^	2
Hospital stay, days	40

Data are expressed as mean ± standard deviation, number (%), or median (25–75% interquartile range)

*AoV* aortic valve, *AVR* aortic valve replacement, *CABG* coronary artery bypass grafting, *ICU* intensive care unit, *MV* mitral valve, *MVP* mitral valve repair

^a^Either primary operation or re-operation

^b^Including 2nd ICU stay (10 patients)

### Microbiologic data

Data about the causative microorganism was available for all but six patients (Tab. 3). Four of these patients had a negative blood or tissue culture, and the medical records of two patients were incomplete in this respect. The most common cultivated organisms were Staphylococci species (28%), Streptococci species (28%) and Propionibacterium species (14%). No significant effect of the offending microorganisms on survival time could be found for these three groups. However, in the remaining group of patients, a significant association was found between the offending microorganism and survival: (*χ*^2^(1) = 8.105, *p* = 0.004). This latter group consisted of eleven patients infected with eight different types of bacteria. Within this group, there were three cases of *Enterococcus faecalis* and two cases with different Pseudomonas species. All other patients were infected by different bacteria.

**Table 3:** Microorganisms overview

Offending microorganisms	*N* (%)
Streptococci	19 (28)
Staphylococci	19 (28)
Propionibacteria	14 (20)
Other	11 (16)
Unknown	2 (3)
Tissue and/or blood culture negative	4 (6)

### Postoperative complications:

Due to heart block, 23 patients required implantation of a permanent pacemaker; one patient received palliative care, hence a pacemaker was contraindicated. Five patients (7%) had a stroke (CVA), whereas nineteen patients (28%) had pulmonary complications (Tab. 4). Ten patients (15%) required a re-exploration for bleeding. During the follow-up, eight patients (12%) suffered from reinfection, seven of which affected the aortic valve and one the mitral valve. Seven of these patients underwent re-intervention, whereas one passed away before re-intervention was performed. Three of the patients who underwent re-intervention died within 30 days of surgery. Freedom from recurrent endocarditis was 83.5 ± 13.3% at 10 years.

**Table 4:** Postoperative complications & long-term outcome

Complication	*N* (%)
Re-exploration for bleeding	10 (15)
Pulmonary complications	19 (28)
Cerebrovascular accident	5 (7)
30-day mortality	9 (13)
120-day mortality	11 (16)
Overall mortality	25 (36)
Pacemaker implantation	23 (33)
Reinfection	8 (12)
Re-intervention	7 (10)

**Table 5:** Comparison of studies concerning management and outcomes of aortic valve endocarditis complicated by periannular abscess

Reference	*n*	NVE vs PVE	Prosthesis Bio vs Mech	Offending Microorganisms	Mortality	Freedom from re-endocarditis	Freedom from re-operation	Survival	Comment
Present study	69	51–49%	28–72%	Staphylococci: 28% Streptococci: 28% Enterococci: 4%	13% (30-day) 16% (120-day) 36% (overall)	83.5 ± 13.3% (10 years)	85.9 ± 10.6% (10 years)	69 ± 12% (5 years) 56 ± 14% (10 years)	
Kirali (2016) [28]	27	78–22%	7–36%	Staphylococci: 37% Streptococci: 15% Enterococci: 11%	22% (hospital) 26% (30-day)			63 ± 6% (5 years) 59 ± 7% (10 years)	Inclusion ARR patients
Takahashi (2013) [18]	25	68–32%	36–64%	Staphylococci: 48% Streptococci: 16% Enterococci: 12%	20% (30-day)	100% (3 years)	100% (3 years)	80 ± 8% (3 years)	Patients with LV-Ao discontinuity
Leontyev (2012) [16]	172	56–44%	82–11%	Staphylococci: 40% Streptococci: 23% Enterococci: 15%	25% (30-day)	80 ± 4% (5 years)		50 ± 4% (5 years)	Ao allograft 7.5%
David (2007) [13]	135	51–49%	41–49%	Staphylococci: 46% Streptococci: 26% Enterococci: 4%	16% (operative)	88 ± 3% (5 years) 82 ± 4% (10 years) 82 ± 4% (15 years)	96 ± 2% (5 years) 84 ± 5% (10 years) 72 ± 9% (15 years)	71 ± 4% (5 years) 57 ± 5% (10 years) 43 ± 6% (15 years)	Ao allograft 10%
Naqvi (2005) [17]	45	47–53%		Staphylococci: 56% Streptococci: 9% Enterococci: 16%	31% (hospital)			62% (1 year)	37 (82%) patients had surgery
Anguera (2005) [4] ^a^	67	60–40%		Staphylococci: 36% Streptococci: 28% Enterococci: 12%	19% (hospital)				IE with abscess vs without
Knosalla (2000) [15]	65	72–28%	5–23%	Staphylococci: 40% Streptococci: 35% Enterococci: 12%	24% (30-day) 89% (allograft)	72% (11 years) 97% (allograft)	73% (11 years) 90% (allograft)	65% (11 years) 82% (allograft)	Ao allograft 72%
d’Udekem (1996) [14]	70	49–51%	43–51%	Staphylococci: 53% Streptococci: 30% Enterococci: 3%	13% (operative)	76 ± 10% (8 years)	4.3%	64 ± 8% (8 years)	Ao allograft 6%

*Ao* aorta, *ARR* aortic root replacement, *IE* infective endocarditis, *LV* left ventricle,* NVE* native valve endocarditis, *PVE* prosthetic valve endocarditis

^a^ Multicentre study

### Long-term survival

Fourteen patients died during follow-up. Five-year and 10-year survival was 69.4 ± 12.0% and 55.7 ± 14.3%, respectively. It was found that patients who had PVE, and thus underwent a re-operation, had significantly lower survival time than patients with a native valve endocarditis (NVE) (*χ*^2^(1) = 5.472, *p* = 0.019). On the other hand, patients with a bicuspid valve were found to have a higher survival time than those with a normal valve (*χ*^2^(1) = 5.083, *p* = 0.024). Mechanical valves were implanted in 50 patients (72%) of this group. It was found that patients who received a mechanical valve had significantly higher survival time than those that received biological valves (*χ*^2^(1) = 7.049, *p* = 0.008). Out of the preoperative comorbidities reported in Tab. 1, three were found to have a significantly negative effect on survival time. These were diabetes mellitus (*χ*^2^(1) = 4.878, *p* = 0.027), peripheral vascular disease (*χ*^2^(1) = 5.276, *p* = 0.022), and transient ischaemic attack/CVA (*χ*^2^(1) = 10.714, *p* = 0.001). Table 5 shows a comparison of earlier studies concerning the management and outcome of aortic valve endocarditis complivated by periannular abscess.

## Discussion

This 20-year retrospective observational study was conducted to investigate the long-term outcomes of patients with aortic infective endocarditis complicated by periannular abscess formation, treated with gentamicin filling, annular reconstruction, and aortic valve replacement. Overall mortality, reinfection, and re-intervention were the primary endpoints of this study. This technique has yielded an acceptable long-term outcome in terms of survival and rate of re-intervention.

Aggressive surgical treatment is crucial for treating infective endocarditis effectively as it remains associated with a high complication and mortality rate [2, 3]. Early mortality has been reported to be twice as high in patients with infective endocarditis and annular abscess formation and is associated with more late complications [12]. According to the presented data, an acceptable long-term survival was achieved considering the severity of the disease in this patient category. The overall mortality in the present series was 25 patients (36%), with a 30-day mortality of 9 patients (13%) and 120-day mortality of 11 patients (16%). Other studies have reported a 30-day mortality rate for surgery for paravalvular abscess ranging from 20 to 31% [4, 13, 15–20]. This excludes studies using only homografts.

Radical debridement of abscess cavities is a universally accepted procedure in cardiac surgery and is of primary importance in active infective endocarditis. In most cases, reconstruction of the aortic annulus is required using a pericardial patch, thereby closing the abscess cavity. This procedure is contradictory to the typical treatment of abscess cavities in which frequent rinsing is required. Topical administration of antibiotics, such as vancomycin and gentamicin, is usually applied to sternotomy wounds as prophylaxis or after deep sternal infections [21–23]. In the present series, a gentamicin-treated sponge was inserted in the abscess cavity before closure and reconstruction of the annulus. Other studies have reported the application of antibiotic-impregnated biological sealant into abscess cavities [18, 19, 24]. Local administration of antibiotics is advantageous as it results in high local concentrations without reaching toxic serum levels that could lead to renal damage and other complications [22].

The most common offending microorganisms cultivated in the present study were similar to other reports [4, 13–18, 20]. The predominant organisms causing infective endocarditis and abscess formation were species of Staphylococci (28%) and Streptococci (28%) followed by Propionibacteria (14%). *Staphylococcus aureus* (*S. aureus*) is the primary cause of infective endocarditis [25], especially in PVE, with a worse long-term survival observed in patients with PVE caused by *S. aureus* compared with other organisms [26]. This is also the case in NVE, as a large merged database demonstrated [27]. Despite a significant association being found between survival and other offending microorganisms in 11 (16%) of the patients, this limited patient cohort was underpowered to adjust survival for other variables. Generally, we did not find a correlation between causative microorganisms and the mortality in our study population due to the diversity of bacteria found.

The mean five-year and 10-year survival rate in our study are comparable to previous studies suggesting that surgical treatment for infective endocarditis complicated by periannular or paravalvular abscess formation corresponds with a declining survival rate with increasing follow-up time [5, 13, 28]. Kirali and colleagues presented a five-year and 10-year survival rate of 62.9 ± 6.4% and 59.2 ± 7.2%, respectively [28]. A possible explanation for this slight difference compared to the current series can be their smaller patient population (*n* = 27) or their inclusion of patients undergoing aortic root replacement. The study conducted by David *et al.* [13] in patients with paravalvular abscess formation reported a comparable five-year and 10-year survival rate of 71 ± 5% and 57 ± 5%, respectively. However, abscess cavities were left untreated in their study and the patient population was younger than our series (51 ± 16 vs 58 ± 15 years). The same study demonstrated worse outcomes of surgery on PVE vs NVE: long-term 10-year survival in patients with PVE was 52 ± 7% vs 62 ± 6% for NVE [13]. In our patient group, 10-year survival was found to be 43.2 ± 20% for PVE vs 68.8 ± 19% for NVE, suggesting that survival was indeed significantly lower in PVE than in NVE. The number of mortality events (*n* = 25) was too small to adjust survival for other variables. These results are in accordance with earlier research findings inferring worse early and late outcomes in PVE as it is frequently associated with periannular abscess, increasing the complexity of surgical treatment [4, 5, 28].

The findings of the present series show that the utilised approach resulted in successful eradication of infective endocarditis in the majority of patients. Freedom from recurrent endocarditis was 83.5 ± 13.3% at 10 years. These results are similar to earlier studies reporting late (8–11 years) freedom from infective endocarditis recurrent rates of 72–82% [13–15]. Earlier reports have noted that aortic root replacement has been considered a successful alternative technique for the management of aortic valve infective endocarditis with periannular abscess. According to these reports, this technique has a low reinfection rate but a progressive elevated risk of valve deterioration [10]. An allograft is associated with high rates of early calcification and limited availability [29], although superior capabilities against recurrent infective endocarditis have been reported. Sabik *et al.* [30] reported favourable results in 103 patients with PVE complicated by aortic root abscess. Only four patients had recurrent endocarditis resulting in a 10-year freedom of recurrent endocarditis of 95%. Yankhah and colleagues [20] demonstrated a comparable 10-year freedom of recurrent endocarditis of 92% using antibiotic-permeable cryopreserved allograft compared to freehand aortic valve replacement in 160 patients with periannular abscess. Although most surgeons believe in better outcomes with allografts regarding recurrent infection, we believe that choice of material is not determinative for good results. The most crucial part of the surgical procedure remains radical debridement and attachment of the prosthetic/allograft to strong, healthy and, most importantly, non-infected tissue.

The primary limitation of this series is the relatively small patient population and the absence of a control group. However, compared to previous single-centre studies with a similar patient population, our study is among the largest with regard to the population size. Moreover, the choice of gentamicin was not based on the susceptibility of the offending microorganism. On the other hand, one of the strengths of this study is the relatively long follow-up with relevant endpoints. Future research may consider including data from other hospitals and should investigate the impact of other types of antibiotic sponges on long-term outcomes of infective endocarditis of the aortic valve complicated by periannular abscess formation.

In conclusion, surgery for advanced aortic valve infective endocarditis with periannular abscess remains a high-risk procedure with high mortality and morbidity. Radical resection of the infected tissue and proper treatment of abscess cavities is pivotal. Compared with other techniques, relatively good results were achieved in this series by filling abscess cavities with gentamicin-treated sponges and reconstructing the annulus with bovine pericardium patches. In view of the severity of the disease, long-term results are satisfactory with this surgical approach.
